# Severe Persistent Hyperkalemia with Electrocardiogram Changes in a Patient with Hyperaldosteronism

**DOI:** 10.7759/cureus.11358

**Published:** 2020-11-06

**Authors:** Amr Elmoheen, Larissa Michael Mishreky, Shadi Albeiruti, Rabab Helmi Elanani, Aftab Azad

**Affiliations:** 1 Emergency Department, Hamad Medical Corporation, Doha, QAT; 2 Emergency Medicine, Qatar Health - College of Medicine, Qatar University, Doha, QAT

**Keywords:** ecg changes, hemodialysis, hyperkalemia, hyperaldosteronism

## Abstract

A 62-year-old female presented to the emergency department (ED) with fatigue and generalized body weakness for the last three days. Upon arrival, initial ECG showed wide complex tachycardia with sine waves and a heart rate (HR) ranging between 100-170 bpm. She was otherwise vitally stable. The patient had a past medical history of hyperaldosteronism, type 2 diabetes mellitus (DM), chronic kidney disease (CKD) with microalbuminuria, and hypertension. She also had a history of cerebrovascular accident (CVA) and residual left-sided weakness more pronounced in the upper limb. Initial venous blood gas (VBG) analysis showed a potassium level of more than 10 mmol/L, chloride 114 mmol/L, bicarbonate 9 mmol/L, sodium 135 mmol/L, and pH of 7.1. Treatment for hyperkalemia was started immediately with calcium gluconate 1 gm that effectively narrowed her QRS complex and normalized her ECG. Salbutamol nebulization, glucose/insulin infusion, and calcium polystyrene syrup were given. Later, she was started on 100 mg sodium bicarbonate infusion, and Foley’s catheter was inserted to follow urine output (UOP) strictly. However, she did not show a decrease in serum potassium levels. Then the patient underwent hemodialysis for two hours. Her first potassium reading after hemodialysis was 5.2 mmol/L. The purpose of this case report is to emphasize the importance of hemodialysis in patients with persistent severe life-threatening hyperkalemia.

## Introduction

Hyperkalemia is caused by a range of different conditions, such as chronic kidney disease (CKD) and crush injuries, or it can be medication-induced too. Mild hyperkalemia can often be asymptomatic. However, severe hyperkalemia is a life-threatening condition that requires emergency treatment. It can lead to dangerous cardiac arrhythmias, muscle weakness, or muscle paralysis [1]. The aggressiveness of treatment greatly depends on the potassium level. Management protocol for hyperkalemia includes stopping any exogenous potassium intake, giving a calcium salt like calcium chloride or calcium gluconate, and also starting a glucose/insulin infusion. Beta-2 adrenergic drugs also aid in shifting potassium inside the cells. Sodium bicarbonate can be given to patients with metabolic acidosis, and diuretics can help in excreting potassium in the urine [2]. The potassium level is continuously monitored while administering all these drugs. Renal replacement therapy (RRT) is required for severe hyperkalemia and those not responding to drug therapy. It can clear 50-80 mmol of potassium in a four-hour dialysis session [3]. Patients with CKD are prone to hyperkalemia as kidneys are involved in potassium clearance. The presentation and outcome of hyperkalemia can be variable in such patients [4]. Also, hyperkalemia can occur in patients getting treated for hyperaldosteronism [5].

This report describes a unique presentation of life-threatening hyperkalemia in a patient with CKD and hyperaldosteronism. The patient had persistent severe hyperkalemia even after multiple treatments and only responded to hemodialysis.

## Case presentation

A 62-year-old female presented to the emergency department (ED) with the complaint of fatigue and generalized body weakness for the last three days. The patient denied any chest pain, palpitation, shortness of breath, fever, decreased urine output (UOP), recent diarrhea, dysuria, nausea, vomiting, or abdominal pain. Upon arrival, she was otherwise vitally stable with a blood pressure of 128/101 mmHg, 37.8 °C body temperature, and a Glasgow Coma Scale (GCS) of 15.

**Figure 1:**
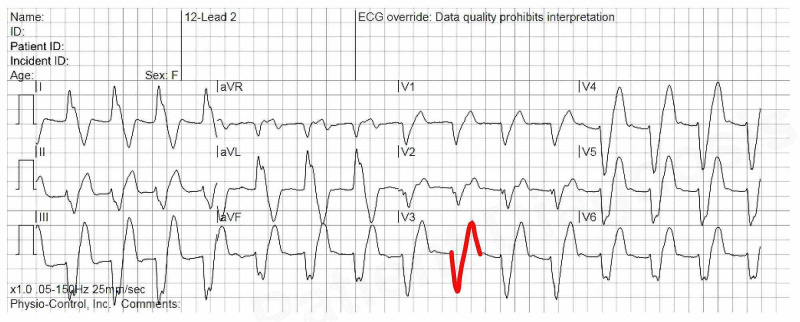
Electrocardiogram (ECG) shows a wide complex tachycardia with sine waves (red color)

Initial Electrocardiogram (ECG) showed wide complex tachycardia with sine waves and a heart rate (HR) ranging between 100-170 bpm (Figure 1).

The patient had a past medical history of type 2 diabetes mellitus (T2DM), CKD with microalbuminuria, hypertension, cerebrovascular accident (CVA) with left-sided weakness more pronounced in the upper limb, and hyperaldosteronism. Her previous investigations showed a baseline creatinine of 130 mmol/L 1.5 years ago. She was taking an angiotensin-converting enzyme (ACE) inhibitor - perindopril 10 milligrams (mg) daily for hypertension and metformin 500 mg twice daily for T2DM. She was a diagnosed case of hyperaldosteronism since 2016; she was diagnosed after a period of persistent hypertension and hypokalemia. Her diagnosis was made by magnetic resonance imaging (MRI) which showed a bulky left adrenal gland with minor morphologic changes, elevated aldosterone (1,510.00 picomoles per liter (pmol/L); normal range 48-644 pmol/L) and aldosterone-renin ratio (ARR) value was higher than the cutoff. For hyperaldosteronism, she was treated medically with potassium chloride (KCL) tablets (600 mg BID) and spironolactone (500 mg once a day).

Investigations

Initial venous blood gas (VBG) analysis showed a potassium level of more than 10 mmol/L, chloride 114 mmol/L, bicarbonate 9 mmol/L, sodium 135 mmol/L, and pH of 7.1 (Table 1). Her kidney function test showed elevated urea (21 mmol/L) and creatinine (289 mmol/L). Complete blood count (CBC) and inflammatory markers, including C-reactive protein (CRP), lactate, and procalcitonin, were all within normal ranges.

**Table 1:** Venous blood gas (VBG) mmHg: millimeters of mercury, mmol/L: millimoles per liter

	Value	Normal Range
pH	7.174	7.320-7.420
Partial Pressure of Oxygen (PO2)	42 mmHg	25-40
Partial Pressure of Carbon Dioxide (PCO2)	29 mmHg	41-51
Sodium (Na)	137 mmol/L	135-145
Potassium (K)	9.7 mmol/L	3.5-5.0
Chloride (Cl)	121 mmol/L	96-110
Bicarbonate (HCO3)	12.0 mmol/L	21.8-26.2

Treatment

**Figure 2:**
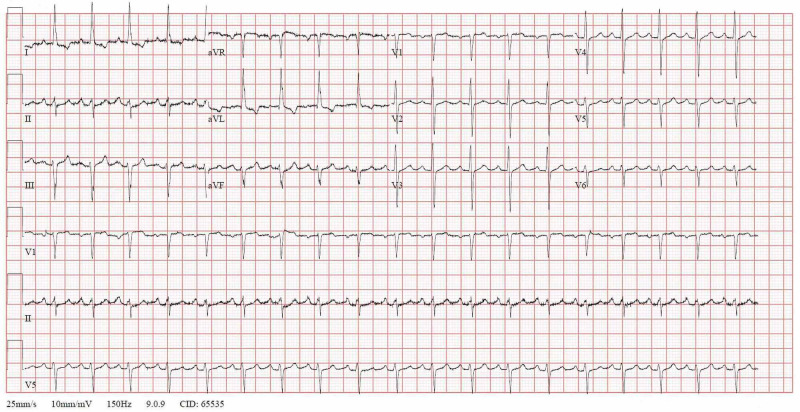
Electrocardiogram (ECG) shows a narrow complex tachycardia and disappearance of the sine waves

Treatment for hyperkalemia was started immediately with intravenous administration of calcium gluconate 1 gm slowly over 10 minutes that effectively narrowed her QRS complex and normalized her ECG by restoring the normal gradient between threshold potential and resting membrane potential (Figure 2).

Salbutamol nebulization was also given twice simultaneously with the intravenous infusion of 50 ml of dextrose 50% along with 10 units of regular insulin. Calcium polystyrene syrup (50 gm) was also given orally.

Outcome

Repeated VBG analysis showed persistently elevated potassium (more than 6.9 mmol/L). She was given two more doses of each, glucose/insulin and salbutamol, again. VBG was repeated after these doses and showed a potassium level of above 7 mmol/L.

Right after seeing no improvement in potassium level, she was started on 100 mg sodium bicarbonate infusion, and Foley’s catheter was inserted to follow UOP strictly. It drained 300 ml of clear urine on insertion. However, she continued to have high potassium readings despite the repeated lines of treatment.

At this point, the nephrologist was involved in performing urgent hemodialysis as she was not improving. Her VBG continued to show metabolic acidosis, and the potassium level did not decrease below 6.8 mmol/L. After written consent from the patient was obtained, ultrasound-guided central venous line insertion was carried out in the right femoral vein, and the patient underwent hemodialysis for two hours. Her first potassium reading after hemodialysis was 5.2 mmol/L.

The patient was discharged home after changing her antihypertensive medication to oral verapamil 120 mg daily. The patient remained asymptomatic and did not develop other attacks of hyperkalemia. She was on regular follow-up with the endocrinology and nephrology out-patient clinics.

## Discussion

In our case report, severe life-threatening level of hyperkalemia causing ECG changes in this patient who did not have surgical adrenalectomy for hyperaldosteronism could be multifactorial.

The most prominent factor was the medications she was taking. The patient was taking KCL 800 mg BID for about the last 1.5 years, and she was not following up with her primary physician during this period. Also, no follow up lab investigations like serum electrolytes were carried out during all of this period. Exogenous potassium coming from diet or supplements can cause hyperkalemia, and it is more pronounced in patients with CKD [6], which was also the case with our patient.

In addition to potassium supplements, the patient was also taking potassium-sparing diuretics spironolactone and perindopril, and both of them can also contribute to raising serum potassium level [7]. Investigations also showed an increase in serum creatinine from 133 mmol/L to 289 mmol/L during the same period that reflects the progression of CKD. However, it is not clear if it had played a major role in her presentation with hyperkalemia. CKD is associated with hyperkalemia as kidneys play a more role in potassium clearance [8]. In this particular case, the role of CKD in the patient’s acute presentation is unclear as the progression of underlying CKD was not massive, and the patient was not in a state of end-stage renal disease (ESRD) given the good UOP and her estimated glomerular filtration rate (eGFR) was 15 ml/min/1.73m2. Therefore, the patient’s acute presentation with severe hyperkalemia seems not to be associated with her CKD. Although the patient also had metabolic acidosis at presentation, it is one of CKD’s most common manifestations [9].

Another contributing factor to her severe hyperkalemia can be her previous stroke. She had residual left-sided weakness from the old CVA episode that could have contributed to her high potassium level. Her muscle weakness caused by stroke led to muscle wasting and immobility that can increase potassium levels [10].

The patient was treated for hyperkalemia as per protocols, and the line of treatments that involved intracellular potassium shift stabilized her initially. Still, it did not solve the main problem, i.e., the high build-up of serum potassium level throughout the last year. The patient only responded to hemodialysis after all the discussed treatment options failed to improve her hyperkalemia.

Other treatment lines that help in the excretion of potassium out of the body, such as sodium polystyrene sulfonate, were started early as well. Still, the effect is significantly delayed and questionable. Such medications treat chronic hyperkalemia in cases where the chances of complications are low and hyperkalemia is mild [11]. Therefore, it was not a very suitable treatment option for severe hyperkalemia with the risk of complications. Furosemide diuretic was also considered early in the management plan, but there was a chance of CKD progression and further elevation of creatinine [12], so it was held away.

Through this case report, we want to emphasize the importance of hemodialysis in case of severe refractory hyperkalemia with the risk of complications in patients with multiple underlying causes of hyperkalemia. If severe hyperkalemia does not respond to standard drug treatment, hemodialysis should be considered.

## Conclusions

Hemodialysis helps treat hyperkalemia in cases where standard treatment does not work. In cases with multiple underlying causes of hyperkalemia, it can be difficult to determine the exact cause of acute presentation with severe hyperkalemia. In such patients who do not respond to drugs that shift potassium intracellularly or excrete it from the body, hemodialysis should be carried out to prevent life-threatening complications of severe hyperkalemia, such as cardiac arrhythmias, especially in patients with ECG changes.
